# Exotic Diseases

**Published:** 1899-02

**Authors:** 


### Exotic Diseases.

[General Orders No. 15.]

Headquarters of the Army,
Adjutant-General's Office,
Washington, D. C., January 20, 1899.

By direction of the Secretary of War, the following orders of the President are published for the information and guidance of all concerned:

Executive Mansion,
Washington, D. C., January 17, 1899.

To prevent the introduction of epidemic disease, it is ordered that the provisions of the act of Congress approved February 15, 1893, entitled "An act granting additional quarantine powers and imposing additional duties upon the Marine Hospital Service," and all rules and regulations heretofore prescribed by the Secretary of the Treasury under that act are to be given full force and effect in the islands of Cuba and Porto Rico, and the following additional rules and regulations are hereby promulgated:

The examination in ports of the islands of Cuba and Porto Rico of incoming and outgoing vessels and the necessary surveillance over their sanitary condition as well as of cargo, passengers, crew, and of all personal effects, is vested in and will be conducted by the Marine Hospital Service, and medical officers of that service will be detailed by the Secretary of the Treasury as quarantine officers at the ports of Havana, Matanzas, Cienfugos, and Santiaga immediately, and at other ports in the islands of Cuba and Porto Rico as soon as practicable or necessary. Quarantine officers shall have authority over vessels, their wharfage and anchorage in infected seaports, in so far as is necessary to prevent the infection of vessels or their personnel, and all vessels, including vessels of the army transport service and merchant and coastwise vessels leaving ports in the islands of Cuba or Porto Rico for the United States or for other ports in the islands of Cuba or Porto Rico, vessels of the United States Navy excepted. Quarantine officers will enforce necessary measures on incoming vessels through collectors of customs at ports of entry, who will not permit entry without quarantine certificates, and bill of health shall not be given to an outgoing vessel unless all quarantine regulations have been complied with. All officers of the army transport service and medical officers of the Army and Marine Hospital Service on duty on army transports will use every precaution to prevent danger of exposure to infection of crews while in ports in the islands of Cuba or Porto Rico.

Since the quarantine service herein provided is for the protection of the islands of Cuba and Porto Rico as well as the protection of the United States against both, the expenses arising therefrom will be charged at present both against the revenues of these islands and the epidemic fund; said expenses will be divided equally against both, payments, however, to be made out of the epidemic fund and reimbursement made thereto from the revenues of the islands of Cuba and Porto Rico.

WILLIAM McKINLEY.

By Command of MAJOR-GENERAL MILES.

H. C. CORBIN, Adjutant-General.

CORRECTION.

LEGATION OF MEXICO,

*Washington, D. C., January* D, VRTT.

MR. SECRETARY: In pursuance of instructions received from Mr. Mariscal, minister of foreign relations of my country, I have the honor to inform you that No. 47 of the Public Health Reports, issued by the Marine Hospital Service of this country, bearing date of November 25th, last, among its statistics of infectious diseases in various countries, states that, on September 28th, last, there was a case of yellow fever at Jimenez, State of Tamaulipas, in the United States of Mexico, and that, a suitable investigation having been made, the fact has been established that neither before nor since September 28th has there been any case of yellow fever in that town. I, therefore, beg you to be pleased, if there is no objection, to cause a suitable correction to be made in the publication in question.

I renew to you, Mr. Secretary, the assurances of my highest and most distinguished consideration.

JOSE F. GODOY.

HON. JOHN HAY.

—*From Public Health Report.*

